# Follicle-stimulating hormone peptide-conjugated nanoparticles for targeted shRNA delivery lead to effective gro-α silencing and antitumor activity against ovarian cancer

**DOI:** 10.1080/10717544.2018.1440667

**Published:** 2018-02-20

**Authors:** Shan-Shan Hong, Ming-Xing Zhang, Meng Zhang, Yi Yu, Jun Chen, Xiao-Yan Zhang, Cong-Jian Xu

**Affiliations:** a Obstetrics and Gynecology Hospital, Fudan University, Shanghai, China;; b Departments of Pharmaceutics, School of Pharmacy, Fudan University, Shanghai, China;; c Department of Obstetrics and Gynecology of Shanghai Medical School, Fudan University, Shanghai, China;; d Shanghai Key Laboratory of Female Reproductive Endocrine Related Diseases, Shanghai, China

**Keywords:** Ovarian carcinoma, targeted therapy, follicle-stimulating hormone, growth-regulated oncogene α, short hairpin RNA

## Abstract

The distinct hormone molecules and receptors, such as follicle-stimulating hormone receptor (FSHR) in ovarian cancer, provide opportunities for more precisely targeted therapy. We previously developed FSHR-mediated nanoparticles and found that FSH peptides on the surface of nanoparticles improved the delivery of short interfering RNA (siRNA) into ovarian cancer cells. However, the high toxicity of the nanoparticles and the transient silencing of the siRNA *in vivo* limited further study. Here, we developed FSH peptide-conjugated nanoparticles with an increased amount of polyethylene glycol (PEG) grafting and encapsulated short hairpin RNA (shRNA) to silence the target gene, growth-regulated oncogene α (gro-α). The nanoparticle complexes exhibited good stability over three weeks. Expression of the target gene, gro-α, was significantly down-regulated by gro-α shRNA-loaded nanoparticles conjugated with FSH peptides (FSH33-G-NP) in FSHR-positive HEY cells. Cell proliferation, migration, and invasion were also inhibited by FSH33-G-NP. Tumor growth was delayed significantly in the mice treated with FSH33-G-NP. No significant loss of body weight or severe toxic effects were observed in any groups. In conclusion, gro-α shRNA-loaded nanoparticles conjugated with FSH peptides overcame the drawbacks of the *in vivo* application of RNAi therapeutics and polymer-based nanocarriers and showed safe antitumor efficacy. Our study might contribute to the application of FSHR-based targeted therapy and imaging in cancer.

## Introduction

Targeted therapy has been developed over the past several years, along with an extensive understanding of molecular and genetic changes in cancer. In contrast to non-specific chemotherapy, targeted therapeutics are designed to recognize or bind molecules specific to cancer cells and not act on normal cells, leading to a reduction in the side effects of antitumor drugs. Various types of targeted therapeutics have been developed, including angiogenesis, signal pathway, and signal enzyme inhibitors. In addition, receptor-mediated drug delivery and RNA-based therapeutics are under further investigation.

Given that ovarian cancer is a heterogeneous disease with complex molecular changes, as well as a reproductive hormone-related disease, the distinct hormone molecules and receptors involved provide opportunities for more precisely targeted therapy (Zhang & Xu, 2011; Engel et al., 2016). Follicle-stimulating hormone (FSH) is secreted by the pituitary gland and acts on ovaries by the FSH receptor (FSHR). FSHRs are found at high levels in reproductive organs and very low levels or not at all in other tissues. Therefore, FSHR is a rational target for receptor-mediated drug delivery in ovarian cancer. We previously developed FSHR-mediated nanoparticles and found that FSH peptides on the surface of nanoparticles have the potential to selectively deliver paclitaxel to ovarian cancer cells expressing FSHR (Zhang et al., 2009; Fan et al., 2014).

RNA interference (RNAi) has been studied as an antitumor therapeutic strategy due to its efficient silencing of target genes, and some of these drugs have entered clinical trials. Despite the high-therapeutic potential, the clinical use of RNAi therapeutics is still hindered by some problems, including poor stability and poor uptake. Thus, polymer-based nanocarriers and lipid-based carriers have been used to encapsulate RNAi drugs to overcome these problems (Barata et al., 2016).

We previously developed a short interfering RNA (siRNA) delivery system consisting of a polyethylene glycol (PEG)-polyethylenimine (PEI) copolymer modified with FSH β 33–53 peptides to improve the stability, circulation time, and delivery efficiency of the system. This system could mediate the highly selective delivery of siRNA into ovarian clear cell carcinoma cells ES-2 (Hong et al., 2013). However, the acute toxicity observed *in vivo* limited our work. The reason for the high toxicity might be that a low amount of PEG grafting leads to polyplex aggregation. The molecular weight and conjugation ratio of PEG on PEI are related to DNA association and polyplex aggregation, considering that PEG conjugation offers colloid stability and biocompatibility for the PEI-DNA complex (Smith et al., 2015). Moreover, our previous study used siRNA for target gene knockdown. However, the silencing effect of siRNA is transient. Short hairpin RNA (shRNA) is a stem-loop RNA that also silences a specific gene via RNAi (Lam et al., 2015). Compared with siRNA, it provides long-lasting gene silencing and comparable efficiency (Gvozdeva et al., 2016).

In this study, to reduce the toxicity and improve the silencing efficiency of the nanoparticle complex, we prepared an FSH peptide-conjugated PEG-PEI copolymer with an increased amount of PEG grafting and encapsulated shRNA to silence the target gene, growth-regulated oncogene α (gro-α), which promotes malignant transformation, tumor growth, and metastatic spread (Yang et al., 2006; Wang et al., 2017).

## Materials and methods

### Immunocytochemistry and immunohistochemistry

Immunocytochemistry and immunohistochemistry were used to detect the expression of FSHR and gro-α in ovarian cancer cells and tissues. HEY human ovarian cancer cells were seeded in 24-well plates and cultured in RPMI-1640 medium supplemented with 10% fetal bovine serum. After fixation, cells were incubated with FSHR antibody (Abcam, Ltd, San Francisco, CA, USA) or gro-α antibody (Abcam Ltd, HK) at 4 °C overnight. Then, the cells were incubated with HRP-conjugated anti-rabbit IgG (Abcam Ltd, San Francisco, CA, USA) for 30 min at room temperature. The staining reaction was performed with diaminobenzidine. The cells were counter-stained with hematoxylin and imaged by light microscopy (Olympus Corporation, Tokyo, Japan). For ovarian cancer tissue, the procedures were the same as the cells, except the tissue samples were subjected to deparaffinization and rehydration before incubation with antibodies.

### Western blot analysis

Western blot was employed to determine the expression of FSHR and gro-α in ovarian cancer cells. The protein extracts were separated by SDS-PAGE and blotted onto a polyvinylidene difluoride membrane. After blocking, the membrane was separately incubated with FSHR antibody or gro-α antibody at 4 °C overnight. Next, the membrane was washed and incubated with HRP-conjugated goat anti-rabbit secondary antibody (Abcam Ltd, HK) for 1 h at room temperature. Specific protein bands were visualized using ECL Western Blotting Substrate (Pierce Biotechnology, Rockford, IL, USA) and imaged by an ImageQuant™ LAS4000 system (GE Healthcare LifeSciences, Marlborough, MA, USA).

### Screening of shRNA sequences targeting gro-α

Four shRNA sequences targeting gro-α were designed and cloned into an expression plasmid, pcDNA^TM^6.2-GW/EmGFP-miR (Invitrogen Corporation, Shanghai, China; Table 1). The shRNA plasmids were then sequenced. Two micrograms of shGro#1, shGro#2, shGro#3, shGro#4, or shGro#4 with DharmaFECT transfection reagent (Thermo Fisher Scientific, Shanghai, China) were added to HEY cells cultured in 24-well plates. The untreated cells and cells treated with shControl were used as the controls. After incubation for 4 h, the medium containing shRNA was replaced. Then, green fluorescent protein was detected as a marker of transfection efficiency by fluorescence microscopy and flow cytometry.

**Table 1.** The sense and anti-sense oligonucleotide sequences of shRNA targeting gro-α.

	Oligonucleotide sequences 5′-3′
shGro#1 (sense)	TGCTGGCTGGCGGATCCAAGCAAAGTTTTGGCCACTGACTGACTTTGCTTGTCCGCCAGC
shGro#1 (anti-sense)	CCTGGCTGGCGGACAAGCAAAGTCAGTCAGTGGCCAAAACTTTGCTTGGATCCGCCAGCC
shGro#2 (sense)	TGCTGTTTCCGCCCATTCTTGAGTGTGTTTTGGCCACTGACTGACACACTCAAATGGGCGGAAA
shGro#2 (anti-sense)	CCTGTTTCCGCCCATTTGAGTGTGTCAGTCAGTGGCCAAAACACACTCAAGAATGGGCGGAAAC
shGro#3 (sense)	TGCTGCAAGCTTTCCGCCCATTCTTGGTTTTGGCCACTGACTGACCAAGAATGCGGAAAGCTTG
shGro#3 (anti-sense)	CCTGCAAGCTTTCCGCATTCTTGGTCAGTCAGTGGCCAAAACCAAGAATGGGCGGAAAGCTTGC
shGro#4 (sense)	TGCTGTATAATAGGACAGTGTGCAGGGTTTTGGCCACTGACTGACCCTGCACAGTCCTATTATA
shGro#4 (anti-sense)	CCTGTATAATAGGACTGTGCAGGGTCAGTCAGTGGCCAAAACCCTGCACACTGTCCTATTATAC
shControl (Negative control) (sense)	TGCTGAAATGTACTGCGCGTGGAGACGTTTTGGCCACTGACTGACGTCTCCACGCAGTACATTT
shControl (anti-sense)	CCTGAAATGTACTGCGTGGAGACGTCAGTCAGTGGCCAAAACGTCTCCACGCGCAGTACATTTC

### Quantitative real-time PCR (qRT-PCR)

Total RNA was isolated from ovarian cancer cells to detect the mRNA levels of the gro-α gene. Briefly, 1 µg of total RNA was reverse-transcribed with a PrimeScript RT Reagent Kit according to the manufacturer's protocol (TAKARA Bio Inc., Shiga, Japan). qRT-PCR was performed using SYBR Premix Ex Taq™ II (TAKARA Bio Inc., Shiga, Japan), and PCR-specific amplification reactions were conducted in an ABI PRISM 7900 system (Applied Biosystems, Foster City, CA, USA). The primers for detecting the gro-α gene were 5′-GAAAGCTTGCCTCAATCCTG-3′ (forward) and 5′-CACCAGTGAGCTTCCTCCTC-3′ (reverse). The primers for the GAPDH gene were 5′-GCACCGTCAAGGCTGAGAAC-3′ (forward) and 5′-TGGTGAAGACGCCAGTGGA-3′ (reverse). Relative expression levels were calculated using the 2^−ΔΔCT^ method.

### Preparation and characterization of shRNA-loaded nanoparticles

The plasmid expressing shGro#4 was loaded into nanoparticles and conjugated with FSH β 33-53 peptides (YTRDLVYKDPARPKIQKTCTF; China Peptides Co. Ltd., Shanghai, China). Branched PEI (MW 25,000 Da; Sigma Aldrich Co., St. Louis, MO, USA) and Maleimide PEG NHS (MAL-PEG; MW 3,500 Da; JENKEM TECHNOLOGY, Beijing, China) were used. For FSH peptide modification, FSH β 33-53 peptides and MAL-PEG were mixed and magnetically stirred for 6 h. Then, MAL-PEG conjugated with or without FSH peptides was mixed and stirred together with PEI for 24 h. The products, PEG-PEI and FSH-PEG-PEI copolymers, were used to condense the plasmid DNA expressing shGro#4. The feeding molar ratio of PEG:PEI amine was 1:20, and that of FSH peptides:PEG was 4:1.

The grafting of FSH peptides, PEG, and PEI was identified by ^1 ^H nuclear magnetic resonance (NMR) spectroscopy in deuterium oxide. A gel retardation assay was used to detect the combination of the plasmid DNA and PEG-PEI complex at different molar ratio of nitrogen from PEI to phosphate from pDNA (N/P) ratios. A transmission electron microscope (JEOL Ltd, Tokyo, Japan) was used to examine the morphology of the material. The particle size and zeta potential were detected once a week for three weeks using a Malvern Zetasizer Autosizer 4700 (Malvern Instruments, Ltd., Malvern, UK).

### Cell viability assay

Cell viability was determined by CCK-8 assay according to the manufacturer's protocol (Dojindo Laboratories, Kumamoto, Japan). Cells cultured in 96-well plates were treated with gro-α shRNA-loaded nanoparticles conjugated with or without FSH peptides and gro-α shRNA plasmid for 24 h, 48 h, 72 h, and 96 h. All groups were adjusted to a final equivalent gro-α shRNA concentration of 1.5 μg/μl. The untreated cells were used as the control. After incubation, 10 μl of CCK-8 solution was added, and the OD values were measured at 450 nm.

### Migration and invasion assay

Cells were treated in 24-well plates as described above for 24 h. The final equivalent gro-α shRNA concentration was 3 μg/μl. Then, 1 × 10^4^ cells were collected and seeded into the upper chambers of the 8.0-μm pore size cell culture inserts. Uncoated inserts (Millipore Corporation, Bedford, MA, USA) and inserts coated with Matrigel (BD BioSciences, San Jose, CA, USA) were used for the migration and invasion assays, respectively. The inserts were placed into a 24-well plate with cells and incubated for 24 h. Cells penetrating the surfaces of the inserts were fixed with 4% paraformaldehyde and then stained with hematoxylin. Cell counting was performed under light microscopy.

### *In vivo* treatment

Four- to six-week-old athymic mice (BALB*/*c nu*/*nu, female; Shanghai Laboratory Animal Center, Chinese Academy of Sciences, Shanghai, China) were used to establish the tumor model. Cell suspensions containing 1 × 10^7^ HEY cells were subcutaneously injected into the flank of the mice for tumor formation. When the tumors became palpable, the mice were randomly divided into groups with six mice per group. The mice were administered gro-α shRNA-loaded nanoparticles conjugated with FSH peptides (FSH33-G-NP), FSH-PEG-PEI copolymer without gro-α shRNA (NP-Blank) and saline via the tail vein every four days for six consecutive injections. The dose of the injected drugs was 5 mg/kg body weight. The tumor size was measured by calipers, and tumor volume was calculated as length × width^2^/2. The clinical status and body weight of the mice were also observed for three weeks beginning on the day of the first administration.

### Statistical analysis

Statistical analysis of the data was carried out using either GraphPad Prism 6.0 (La Jolla, CA, USA) or SPSS 16.0 (SPSS Inc., Chicago, IL, USA) software. Data were expressed as the mean ± SD. One-way ANOVA and unpaired two-tailed Student’s *t*-test was applied to identify significant differences. *p < *.05 was considered significant.

## Results

### Expression of FSHR and gro-α in ovarian cancer cells and tissues

**Figure 1.**
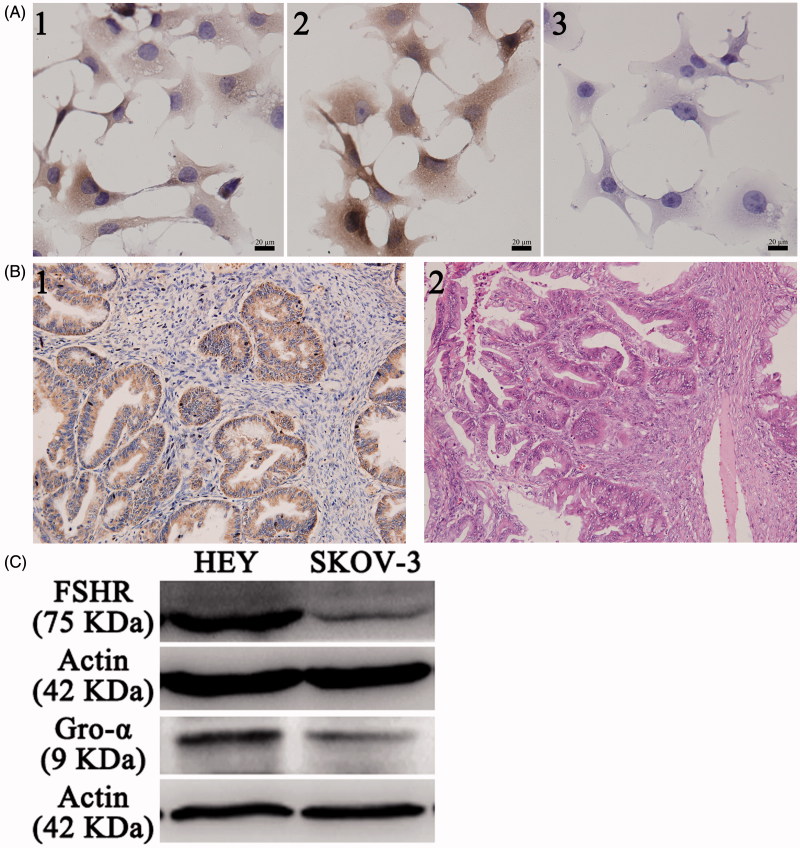
FSHR and gro-α expression in ovarian cancer cells and tissues. (A) FSHR and gro-α expression in ovarian cancer cells by immunocytochemistry. 1, 2 and 3, HEY cells stained with FSHR antibody, gro-α antibody, and negative control. (B) Gro-α expression in human ovarian cancer tissues (200×). 1, gro-α expression; 2, H&E staining. (C) FSHR and gro-α expression in ovarian cancer cells by Western blot.

To screen the appropriate cell lines as cell models, the expression of FSHR and gro-α was detected. As shown in Figure 1(A,C), HEY cells expressed both FSHR and gro-α proteins and were used in subsequent experiments. SKOV-3 cells showed low FSHR expression and positive gro-α expression and were selected as a control, as reported in our previous study (Hong et al., 2013). In addition, gro-α protein expression was also detected in ovarian cancer tissues. The majority of ovarian carcinomas (25 of 30, 83%) showed positive staining for gro-α (Figure 1(B)). Approximately 70% of ovarian cancers express FSHR, as we previously reported (Zhang et al., 2009). Therefore, FSHR was selected as the targeted site, and gro-α was chosen as the targeted gene.

### Screening of shRNA sequences targeting gro-α in ovarian cancer cells

**Figure 2.**
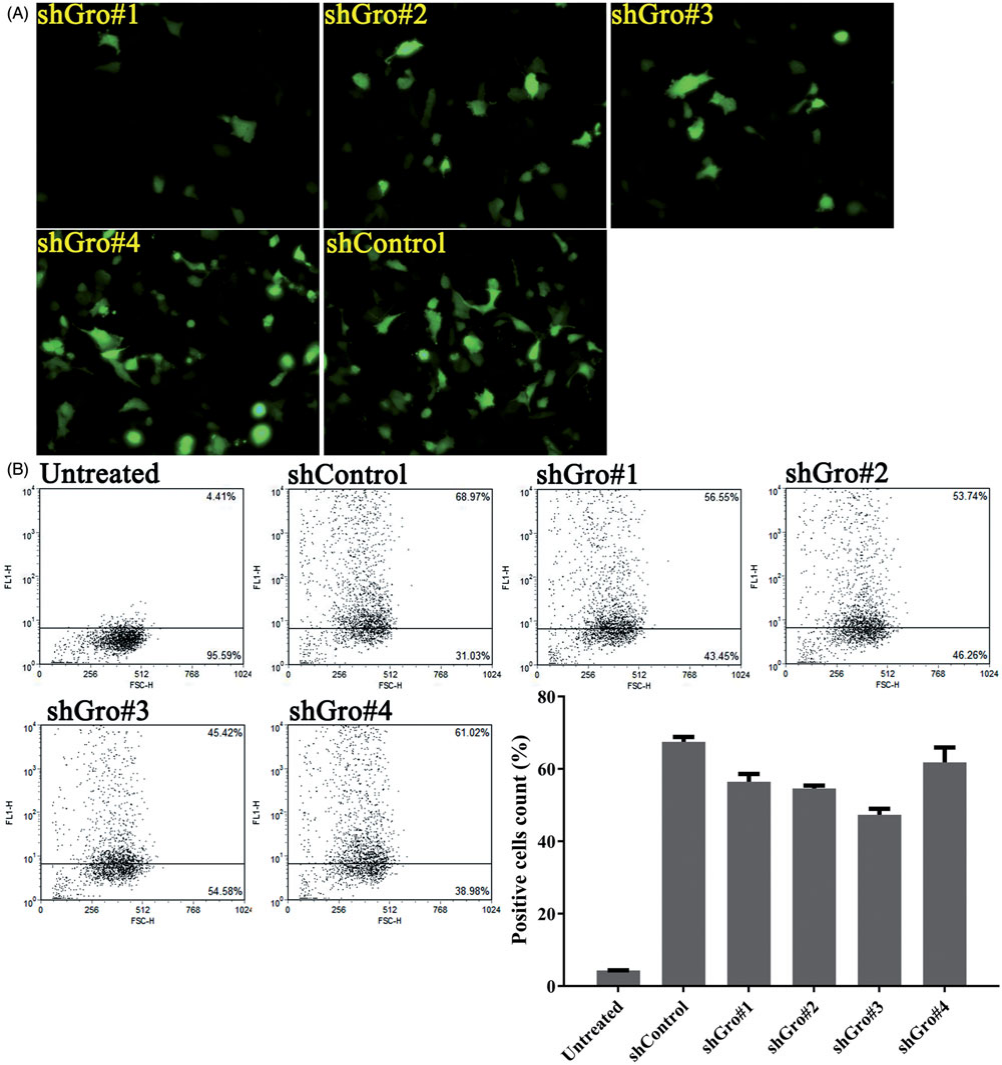
Transfection efficiency of shRNA targeting gro-α in HEY cells. HEY cells were transfected with the plasmids expressing shRNA and green fluorescent protein. Green fluorescent protein was detected as a marker of transfection efficiency by fluorescence microscopy (400×) (A) and flow cytometry (B).

As we previously reported, siRNA targeting gro-α can transiently down-regulate gro-α expression *in vitro* (Hong et al., 2013). To silence gro-α for a long duration, plasmid-based shRNA was utilized in this study. The sense and anti-sense oligonucleotide sequences of four shRNA sequences targeting gro-α are shown in Table 1. The shRNA sequences were screened for transfection efficiency in HEY cells using fluorescence microscopy and flow cytometry. As shown in Figure 2, all four shRNA sequences showed high transfection efficiency in HEY cells. Among them, shGro#4 had the highest transfection efficiency of 61.85%. Thus, shGro#4 was chosen for subsequent experiments.

### Preparation and characterization of shRNA-loaded nanoparticles conjugated with FSH peptides

The plasmid expressing shGro#4 was condensed by PEI, and then the PEI-plasmid was combined with F-PEG (FSH33-G-NP) or PEG (G-NP). The complex containing a low PEG amount was highly toxic to the mice (data not shown). Thus, the amount of PEG in this study was increased by 2.5-fold compared with the PEG/PEI/plasmid complex in our previous study (Hong et al., 2013).

**Figure 3.**
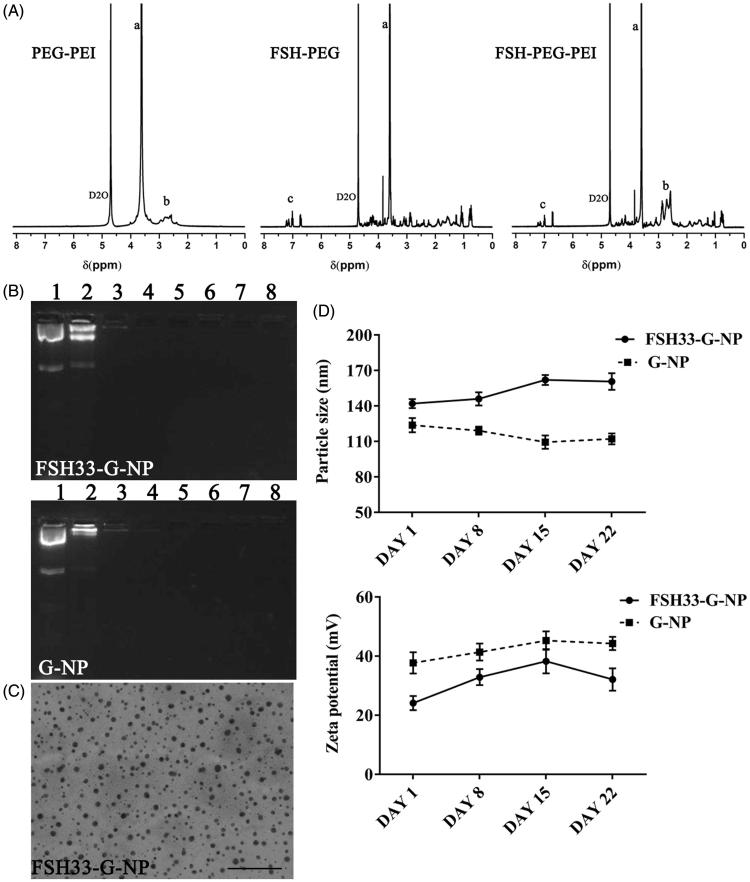
Characterization of nanoparticle complexes. (A) ^1 ^H NMR spectroscopy of copolymers. The distributions of peaks were as follows. a, 3.5–3.6 ppm, PEG (-OCH2CH2-). b, 2.4–3.0 ppm, PEI (-CH2CH2NH-). c, 7.0–7.2 ppm, FSH peptides. The peaks at 4.6–4.7 ppm were chemical shifts from the solvent, D_2_O. (B) The gel retardation assay of FSH33-G-NP and G-NP. Lane 1, plasmid DNA; lanes 2–8, the N/P ratios of 1, 5, 10, 15, 20, 25, and 30. (C) Transmission electron micrograph of FSH33-G-NP. Bar, 500 nm. (D) The particle size and zeta potential of FSH33-G-NP and G-NP in three weeks.

The nanoparticle complexes were characterized by ^1 ^H NMR (Figure 3(A)). The peaks at 4.6–4.7 ppm were chemical shifts from the solvent, D_2_O. The peaks at 2.4–3.0 ppm, 3.5–3.6 ppm and 7.0–7.2 ppm were from PEI (-CH2CH2NH-), PEG (-OCH2CH2-) and FSH peptides, respectively. The spectra of PEG-PEI copolymer conjugated with FSH peptides had extra peaks at 7.0–7.2 ppm compared with PEG-PEI copolymer and extra peaks at 2.4–3.0 ppm compared with FSH peptide-conjugated PEG. These data indicated that PEG, PEI and FSH peptides were grafted together.

The plasmid expressing shRNA was retarded when the N/P ratios were greater than 5 in the gel retardation assay, meaning that the plasmid DNA was completely combined with the nanoparticles (Figure 3(B)). N/P ratio 25 was used in the following experiments. The nanoparticle complexes exhibited spherical shapes (Figure 3(C)). The particle sizes of FSH33-G-NP and G-NP were 142.0 ± 3.8 nm and 123.8 ± 6.0 nm, respectively. The zeta potentials of FSH33-G-NP and G-NP were 24.1 ± 2.4 mV and 37.7 ± 3.6 mV, respectively. Both FSH33-G-NP and G-NP exhibited good stability over three weeks (Figure 3(D)).

### Suppression of gro-α shRNA-loaded nanoparticles on ovarian cancer cells

**Figure 4.**
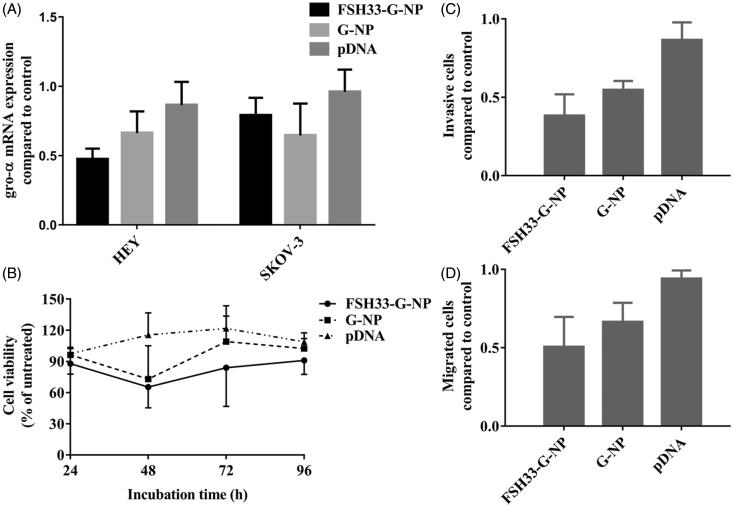
Suppression of gro-α shRNA-loaded nanoparticles on ovarian cancer cells. (A) The levels of gro-α mRNA by qRT-PCR in HEY and SKOV-3 cells treated with FSH33-G-NP, G-NP and gro-α shRNA plasmid. (B) Cell viability as determined by CCK-8 assay in HEY cells treated with FSH33-G-NP, G-NP and gro-α shRNA plasmid for 48 h compared with control. (C) Cell invasion as determined by transwell assay in HEY cells treated with different nanoparticle complexes for 24 h. The inserts coated with Matrigel were used. (D) Cell migration as determined by transwell assay in HEY cells treated with different nanoparticle complexes for 24 h. The uncoated inserts were used.

The down-regulation efficiency of gro-α shRNA-loaded nanoparticles was investigated in ovarian cancer cells. After 48 h of treatment with nanoparticle complexes containing the gro-α shRNA plasmid, cell lysates were obtained, and gro-α mRNA was detected by qRT-PCR (Figure 4(A)). DharmaFECT transfection reagent was not added to the cells treated with gro-α shRNA plasmids. The level of gro-α mRNA in HEY cells was down-regulated to 47.3% and 66.3% of the control level after treatment with FSH33-G-NP and G-NP, respectively. In SKOV3 cells treated with FSH33-G-NP and G-NP, the level of gro-α mRNA was down-regulated to 79.0% and 64.6% of the control level, respectively. Compared with the gro-α shRNA plasmid, gro-α expression was suppressed significantly by FSH33-G-NP in HEY cells. There were no significant differences among the groups in SKOV3 cells.

Thus, the proliferation, migration and invasion of HEY cells were investigated in subsequent experiments. As shown in Figure 4(B), HEY cell proliferation was greatly inhibited by FSH33-G-NP compared with the gro-α shRNA plasmid. The viability of HEY cells treated with FSH33-G-NP and G-NP for 48 h was 65.4% and 73.1%, respectively. Similarly, the invasive and migrated HEY cell numbers were significantly reduced after FSH33-G-NP treatment (Figure 4(C,D)). Thus, gro-α shRNA-loaded nanoparticles conjugated with FSH peptides could inhibit the proliferation, invasion, and migration abilities of FSHR-positive ovarian cancer cells, which may be due to the enhanced down-regulation of gro-α mediated by the nanoparticle complex.

### Antitumor effects of gro-α shRNA-loaded nanoparticles *in vivo*

**Figure 5.**
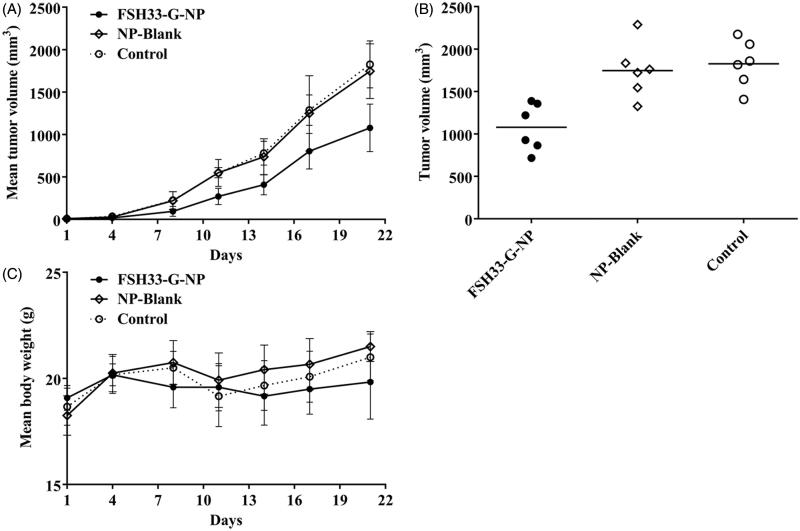
Antitumor effects of gro-α shRNA-loaded nanoparticles *in vivo*. The mice received an intravenous administration of gro-α shRNA-loaded nanoparticles conjugated with FSH peptides (FSH33-G-NP), FSH-PEG-PEI copolymers without gro-α shRNA (NP-Blank) and saline every four days for six consecutive injections (days 1, 4, 7, 10, 13, 16). (A) Tumor volume changes in the mice. (B) Tumor volume at the study end point. (C) Body weight changes of the mice.

To demonstrate the antitumor effects of nanoparticle complexes *in vivo*, a xenograft model of human HEY ovarian cancer cells was established in female BALB/c nude mice. The mice received an intravenous administration of gro-α shRNA-loaded nanoparticles conjugated with FSH β 33-53 peptides. As shown in Figure 5, tumor growth was significantly delayed in the mice treated with FSH33-G-NP compared with those treated with FSH-PEG-PEI copolymer and saline. The inhibitive rate calculated based on tumor volume was 40.9% at the study end point in the FSH33-G-NP group. There was no difference between the FSH-PEG-PEI copolymer (blank nanoparticles) and saline groups. No significant loss of body weight or severe toxic effects were observed in any group, which might be attributed to the PEG grafting. Thus, gro-α shRNA-loaded nanoparticles conjugated with FSH peptides could suppress tumor growth in FSHR-positive ovarian cancer *in vivo*.

## Discussion

Ovarian cancer has the highest mortality rate among gynecological malignancies. Although cytoreductive surgery combined with chemotherapy leads to a better response to initial treatment, the majority of patients still suffer from recurrent disease. The distinct hormone molecules and receptors in ovary and ovarian cancer provide more precise sites for targeted therapy. Here, we used FSHR as the target site and developed FSH peptide-modified nanoparticles to deliver shRNA into ovarian cancer cells. The nanoparticle complexes consisted of FSH β 33-53 peptides, PEG-PEI copolymer, and gro-α shRNA.

Nanoparticles, such as polymer- and lipid-based carriers, have been widely used to deliver RNAi drugs to improve the stability, circulation time, and cell uptake (Debus et al., 2010; Ngamcherdtrakul et al., 2016; Young et al., 2016; Shi et al., 2017). Cationic polymers, such as PEI, are able to strongly condense negatively charged DNA plasmids through electrostatic interactions, leading to high transfection efficiency levels, as well as high toxicity (de Wolf et al., 2007; Sun & Zhang, 2010; Ngamcherdtrakul et al., 2016; Kaczmarek et al., 2017). Thus, PEI as an RNAi drug carrier is often combined with PEG-based polymers because as a neutral and biocompatible polymer, PEG can shield the positive charge on the surface of PEI and improve colloidal stability and blood compatibility (Smith et al., 2015; Ngamcherdtrakul et al., 2016). The amount of PEG grafting and the PEG chain length are related to the ability of PEG to reduce the toxicity of PEI (Park et al., 2005; Zhang et al., 2008; Smith et al., 2015). The chain length and grafting density of PEG also influence siRNA condensation and stability (Mao et al., 2006). When 3% of PEI amines (PEG:PEI amine feeding molar ratio of 1:30) are substituted with MW 5000 Da PEG, the PEG-PEI copolymer has strong DNA association and weak aggregation (Smith et al., 2015). Copolymers with a 1:5 weight ratio of PEG (10 kDa):PEI (25 kDa) also show slightly less toxicity *in vitro* (Feng et al., 2013). In our previous study, a PEG-PEI copolymer with a PEG:PEI amine feeding molar ratio of 1:50 was able to deliver siRNA into ovarian clear cell carcinoma cells with a high efficiency. However, the low amount of PEG grafting on PEI resulted in complex aggregation and high toxicity *in vivo*. Here, the amount of PEG in the copolymer was increased by 2.5-fold, which only caused a slight decrease in the body weight of the mice.

Molecules conjugated onto the surface of nanoparticles are often used as homing devices to mediate active targeting, which can serve as a complementary strategy to the enhanced permeability and retention (EPR) effect and promote the uptake of drugs by targeted cells (Ngamcherdtrakul et al., 2016; Bahrami et al., 2017). Because the expression of FSHR is mainly localized to the ovaries and testes and is lacking in most non-cancerous cells, we used FSHR as the target site for receptor-mediated drug delivery to reduce non-specific uptake. Moreover, FSHR expression is detected in 54.5% of 875 high-grade serous ovarian cancer tissues and is not related to progression-free survival or overall survival (Feng et al., 2016). FSHR has been considered a promising site for targeted therapy or imaging (Hong et al., 2015; Papadimitriou et al., 2016).

In our previous studies, FSHR-binding fragments, including FSH β 33-53 and FSH β 81–95 peptides, facilitated nanoparticle drug delivery into ovarian cancer cells and enhanced the antitumor efficacy of paclitaxel compared with nanoparticles without FSH peptide modification (Zhang et al., 2009; Zhang et al., 2013; Fan et al., 2014). Hence, we used FSHR-mediated nanoparticle carriers to deliver shRNA drugs into FSHR-positive cells, and the results are in agreement with those of previous studies. FSH β 33-53 peptide-conjugated PAMAM dendrimers also exhibit high selective binding and uptake in FSHR-expressing ovarian cancer cells, ovary and oviduct tissues (Modi et al., 2014). T cells expressing FSH subunits can target FSHR and mediate significant therapeutic effects in immunocompetent mice (Perales-Puchalt et al., 2017). Even anti-FSHR immunoreceptors based on FSHR-binding fragments have the potential to deliver T-cell based immunotherapies in ovarian cancer (Urbanska et al., 2015). FSH or FSH peptides have also been used as target moieties in tumor imaging and PET imaging (Lee et al., 2015; Feng et al., 2017; Pan et al., 2017).

The expression of gonadotropin-releasing hormone (GnRH) receptor (GnRHR) in some cancers is higher than in normal tissues. Wu found GnRHR in 88.3% of ovarian cancer tissues (Feng et al., 2016). Various targeted therapeutics using GnRHR as the target site have been developed (Ghanghoria et al., 2016; Li et al., 2017). For example, conjugates of GnRH analogs with chemotherapeutic drugs, delivery carriers, or imaging agents specifically deliver drugs or agents into GnRHR-positive cancer cells rather than GnRHR-negative cells (Shah et al., 2013; Emons et al., 2014; Taratula et al., 2015). It has also been suggested that the specific hormone receptors of ovaries can enhance the precision of targeted therapies against ovarian cancer.

## Conclusion

The gro-α shRNA-loaded nanoparticles conjugated with FSH peptides in this study showed safe antitumor efficacy in mice with FSHR-positive ovarian cancer. The long-lasting gene silencing of shRNA and the increased amount of PEG grafting in nanoparticles helped to overcome the drawbacks of the *in vivo* application of RNAi therapeutics and polymer-based nanocarriers. Our study might contribute to the application of FSHR-based targeted therapy and imaging in cancer.
